# Clinical Characteristics and Management of Triple-Negative Breast Cancer (TNBC) in Jordan: A Retrospective Analysis

**DOI:** 10.7759/cureus.53053

**Published:** 2024-01-27

**Authors:** Hussein Al Husban, Anas Al Rabadi, Ala H Odeh, Kahled Abu Rumman, Feras Alkhawaldeh, Haneen Noures, Mohammad Abo Ashoor, Anas Abu Rumman, Mousa Atmeh, Mohannad Bawaneh

**Affiliations:** 1 Department of General Surgery, Jordanian Royal Medical Services, Amman, JOR; 2 Department of Pathology, Jordanian Royal Medical Services, Amman, JOR; 3 Department of Clinical Oncology, Jordanian Royal Medical Services, Amman, JOR

**Keywords:** neoadjuvant therapy, lymphovascular invasion, jordan, management, clinical characteristics, tnbc, triple- negative breast cancer

## Abstract

Introduction

Triple-negative breast cancer (TNBC) is known for its aggressive nature and poor prognosis. Despite its responsiveness to chemotherapy, TNBC presents challenges in terms of survival, recurrence, and mortality rates, particularly in diverse populations. Limited research in the Middle East hampers comprehensive understanding and tailored management.

Methods

A retrospective study at the King Hussein Medical Center in Jordan between the period 2009 to 2023 explored TNBC patients (n=110) who underwent adjuvant chemotherapy after local excision or modified radical mastectomy (MRM). Data encompassed demographics, clinical variables, and operative details. Statistical analysis employed Wilcoxon and chi-squared tests, examining mortality risks and associations between variables.

Results

Among 110 TNBC patients (mean age 52), 84% underwent MRM, 16% wide local excision and axillary clearance (WLE&AC). Lymphovascular invasion (LVI) was observed in 41%, linked to higher lymph node positivity. Neoadjuvant therapy preceded MRM in 25% of cases. While 75% had grade III tumors, the prevalence of invasive ductal carcinoma was 85%.

Conclusions

This study contributes crucial insights into TNBC characteristics and management in Jordan. Despite limitations such as retrospective design and sample size, the findings underscore the need for tailored interventions in TNBC patients, emphasizing the importance of neoadjuvant therapy and vigilant consideration of LVI status in treatment planning. Future longitudinal research should delve into disease progression and treatment outcomes in diverse populations, facilitating optimized TNBC management strategies.

## Introduction

Triple-negative breast cancer (TNBC) is a category of breast cancer characterized by the absence of estrogen receptor (ER) expression, progesterone receptor (PR) expression, and human epidermal growth factor receptor 2 (HER2) expression, as determined by immunohistochemistry (IHC) [1]. TNBC is renowned for its aggressive nature, often associated with an earlier onset, larger mean tumor size, elevated tumor grade, and occasionally, a greater likelihood of lymph node involvement [2].

TNBC constitutes 15 to 25% of total breast cancer cases, and there is a notably higher mortality rate among African American women, who are recognized to have the lowest survival rates when it comes to this malignancy [3,4]. Although TNBCs are typically associated with a less favorable prognosis concerning breast cancer-specific outcomes, it is noteworthy that a majority of these cancers do not display resistance to chemotherapy. However, patients with this subtype often face an exceptionally grim prognosis, experiencing rapid relapse and a high mortality rate. There are ongoing efforts to develop therapies that specifically target biomarkers associated with TNBC or the basal-like subtype [5,6]. In the management of early-stage TNBC, traditional chemotherapy remains the primary choice for adjuvant systemic treatment in most patients according to the National Cancer Comprehensive Network (NCCN) guidelines.

The comprehensive treatment of breast cancer involves the surgical removal of the tumor and any regional metastatic sites, coupled with localized radiation therapy and chemotherapy, administered in neoadjuvant and/or adjuvant regimens. Surgical techniques vary from radical mastectomy (RM) to organ-preserving procedures, including the option of sentinel lymph node biopsy instead of extensive lymph node dissection when appropriate [7,8]. Radical mastectomy involves the complete removal of breast tissue, muscles, and all axillary lymphatic tissue, with potential complications such as infections, skin necrosis, paresthesias, muscle denervation, damage to rib cartilage, pneumothoraces, and arm lymphedema [9,10]. Studies have demonstrated that breast conservative surgery, such as wide local excision and axillary clearance (WLE&AC), and modified radical mastectomy (MRM), offer an equivalent approach in the early stages of breast cancer. However, these approaches have not been evaluated in previous studies for triple-negative breast cancer (TNBC) [11,12].

There is scarce literature on the prevalence, management, and disease prognosis of TNBC in Jordan and the Middle East. Herein, we aim to describe the clinical, pathological, and prognostic characteristics of a Jordanian sample with TNBC who underwent adjuvant chemotherapy after local excision and modified radical mastectomy.

## Materials and methods

Study design

A retrospective, single-center, observational study was carried out at the King Hussein Medical Center (KHMC) in Jordan between the period 2009 to 2023. Patients with TNBC were recruited to examine the clinical, demographic, and prognostics characteristics of TNBC patients and to analyze how these factors relate to the efficacy of adjuvant chemotherapy and the selection of surgical procedures, specifically modified radical mastectomy (MRM) or breast conservative surgery namely wide local excision and axillary clearance (WLE&AC).

Data collection

Data were collected retrospectively by accessing patients’ hospital records. The following demographic and clinical variables were retrieved: age, surgical approach (WLE&AC or MRM), tumor characteristics such as focality, histology, tumor grade based on histopathology, tumor size, neoadjuvant and adjuvant therapy, number of retrieved lymph nodes, number of positive lymph nodes, lymph-vascular invasion (LVI), and perineural invasion (PNI). Lymph node ratio (LNR) was defined as the number of positive lymph nodes divided by the total number of excised lymph nodes, represented as a percentage (%).

Ethical statement

This research project was approved by the ethical committee at the institutional review board (IRB) at the Jordanian Royal Medical Services (No. 45, Date 9/2023). Informed consent was waived due to the retrospective nature of the study. This study was conducted in accordance with the declaration of Helsinki. All patient data were anonymized and safely stored.

Statistical analysis

Continuous variables were represented as means along with their standard deviations (SD), while categorical variables were summarized using frequencies and percentages. To explore the correlation between demographic, clinical, and operative variables, and the risk of mortality, we employed the Wilcoxon (Mann-Whitney U) test for continuous variables. For categorical variables with a category count of less than 5, we used the chi-squared (X2) test or Fisher's exact test. A significant difference was considered when the p-value was below 0.05. All statistical analyses were conducted using R software (version 4.2.3, Vienna, Austria) [13].

## Results

A total of 110 women with TNBC were recruited. The mean age was 52 (13), and 92 (84%) of them had undergone MRM, while 18 (16%) underwent WLE&AC. Tumor focality was seen on the right side in 56 (52%) of patients, while 51 (48%) had left-sided tumors. Only six patients had sentinel lymph node excision, 28 (25.5%) had neoadjuvant therapy, and 24 (21.8%) had adjuvant chemotherapy. The majority of patients (n = 83, 75%) had grade III tumors, 26 (24%) had grade II tumors, and only one patient had grade I tumors (Table 1).

**Table 1:** Included patient characteristics. ^1^ Mean (SD); n (%). MRM: Modified Radical Mastectomy; WLE&AC: Wide Local Excision and Axillary Clearance; SLN: Sentinel Lymph Node; CTh: Chemotherapy; IMC: Invasive Metastatic Carcinoma; IDC: Invasive Ductal Carcinoma; ILC: Invasive Lobular Carcinoma; LNR: Lymph Node Ratio; LVI: Lymphovascular Invasion; PNI: Perineural Invasion.

Characteristic	N = 110^1^
Age	52 (13)
Neoadjuvant	28 (25%)
Type of surgery	
MRM	92 (84%)
WLE&AC	18 (16%)
Focality	
Left	51 (48%)
Right	56 (52%)
Unknown	3
SLN	
Yes	6 (100%)
Unknown	104
Adjuvant CTh	
Yes	24 (100%)
Unknown	86
Histology	
IMC	15 (14%)
IDC	94 (85%)
ILC	1 (1%)
Grade	
1	1 (0.9%)
2	26 (24%)
3	83 (75%)
Size	
Total LN	19 (9)
Unknown	5
LNR	20 (27)
Unknown	5
LVI	44 (41%)
Unknown	2
PNI	14 (13%)

When comparing patients who underwent WLE&AC vs. MRM, a significant difference in tumors’ focality was seen, in which 11 (73%) of patients who underwent WLE&AC had left-sided tumors, while 52 (57%) of patients who underwent MRM had right-sided tumors (p-value = 0.032). There was a trend towards higher lymph node positivity in patients who underwent MRM, but LNR did not differ significantly between the surgical groups (Table 2). 

**Table 2:** Comparison of patient characteristics based on surgical procedure. ^1^ Mean (SD); n (%). ^2^ Pearson’s Chi-squared test; Wilcoxon rank sum test; Fisher’s exact test. * Statistically significant (p-value < 0.05). MRM: Modified Radical Mastectomy; WLE&AC: Wide Local Excision and Axillary Clearance; SLN: Sentinel Lymph Node; CTh: Chemotherapy; IMC: Invasive Metastatic Carcinoma; IDC: Invasive Ductal Carcinoma; ILC: Invasive Lobular Carcinoma; LNR: Lymph Node Ratio; LVI: Lymphovascular Invasion; PNI: Perineural Invasion.

Characteristic	MRM, N = 92^1^	WLE&AC, N = 18^1^	p-value^2^
Age	53 (13)	50 (11)	0.3
Neoadjuvant	26 (28%)	2 (11%)	0.2
Focality			0.032*
Left	40 (43%)	11 (73%)	
Right	52 (57%)	4 (27%)	
Unknown	0	3	
SLN			
Yes	1 (100%)	5 (100%)	
Unknown	91	13	
Adjuvant CTh			
Yes	13 (100%)	11 (100%)	
Unknown	79	7	
Histology			0.4
IMC	11 (12%)	4 (22%)
IDC	80 (87%)	14 (78%)	
ILC	1 (1%)	0 (0%)	
Grade			0.11
1	1 (1.1%)	0 (0%)	
2	25 (27%)	1 (5.6%)	
3	66 (72%)	17 (94%)	
Size	3.70 (2.42)	2.86 (1.40)	0.12
Positive LN	4.3 (6.3)	1.3 (2.2)	0.045*
Total LN	20 (9)	14 (9)	0.029*
LNR	21 (28)	9 (15)	0.10
LVI	39 (43%)	5 (28%)	0.2
PNI	11 (12%)	3 (17%)	0.7

A total of 44 (40%) patients had LVI, while 64 (58%) were LVI-negative as shown in Table 3.

**Table 3:** Comparison of baseline characteristics based on lymphovascular invasion (LVI). ^1^ Mean (SD); n (%). ^2^ Pearson’s Chi-squared test; Wilcoxon rank sum test; Fisher’s exact test. * Statistically significant (p-value < 0.05). MRM: Modified Radical Mastectomy; WLE&AC: Wide Local Excision and Axillary Clearance; CTh: Chemotherapy; SLN: Sentinel Lymph Node; IMC: Invasive Metastatic Carcinoma; IDC: Invasive Ductal Carcinoma; ILC: Invasive Lobular Carcinoma; LNR: Lymph Node Ratio; LVI: Lymphovascular Invasion; PNI: Perineural Invasion.

Characteristic	LVI, N = 44^1^	Non-LVI, N = 64^1^	p-value^2^
Age	52 (16)	53 (11)	0.8
Neoadjuvant	7 (16%)	21 (33%)	0.049*
Surgery			0.2
MRM	39 (89%)	51 (80%)	
WLE&AC	5 (11%)	13 (20%)	
Focality			>0.9
Left	21 (49%)	30 (48%)	
Right	22 (51%)	32 (52%)	
Unknown	1	2	
SLN			
Yes	1 (100%)	5 (100%)	
Unknown	43	59	
Adjuvant CTh			
Yes	7 (100%)	17 (100%)	
Unknown	37	47	
Histology			0.6
IMC	6 (14%)	9 (14%)
IDC	37 (84%)	55 (86%)	
ILC	1 (2.3%)	0 (0%)	
Grade			>0.9
1	0 (0%)	1 (1.6%)	
2	11 (25%)	15 (23%)	
3	33 (75%)	48 (75%)	
Size	3.89 (1.93)	3.31 (2.54)	0.090
Positive LN	5.8 (6.0)	2.4 (5.5)	<0.001*
Total LN	19 (7)	19 (11)	0.6
LNR	31 (29)	12 (22)	<0.001*
PNI	9 (20%)	4 (6.3%)	0.026*

**Figure 1:**
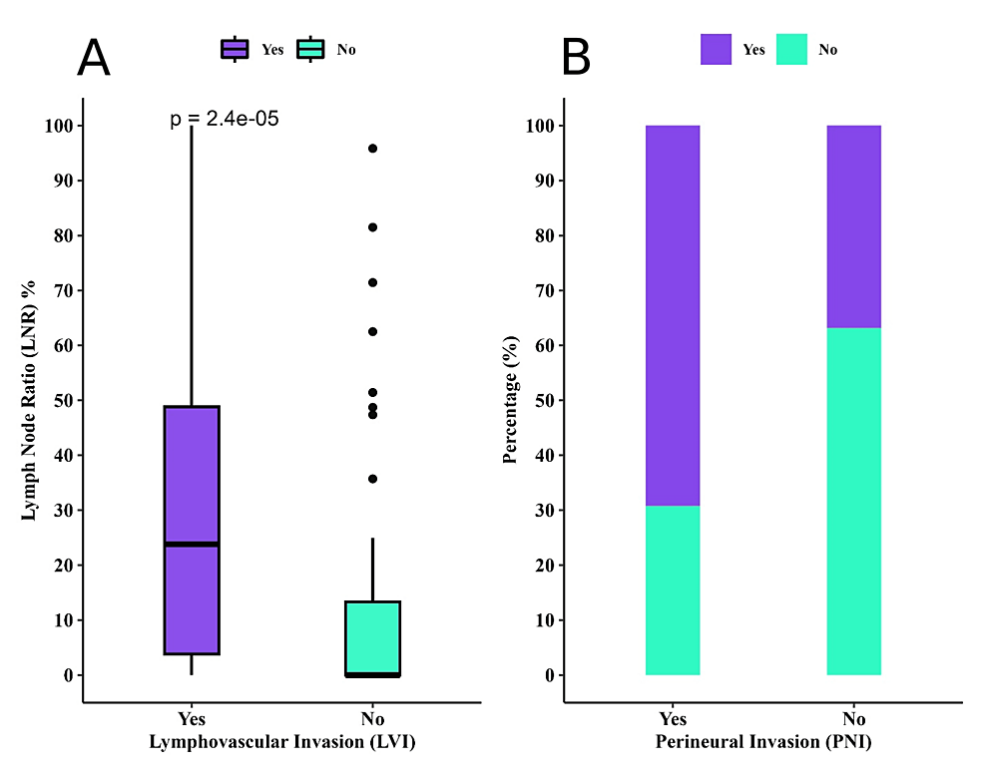
Lymph node ratio rates based on (A) lymphovascular invasion (LVI) and (B) perineural invasion (PNI).

Of the patients with LVI, lymph node positivity was significantly higher compared to LVI-negative patients (mean: 5.8 vs. 2.4, p-value<0.001), in addition to LNR (mean: 31 vs. 12, p-value < 0.001) as shown in Figure 1A. Tumors with LVI showed a trend towards larger sizes than those without LVI (mean: 3.89 vs. 3.31, p-value = 0.090), and a significant association with PNI, in which 9 (20%) of patients with LVI had PNI as shown in Figure 1B.

## Discussion

Breast-conservation therapy such as WLE&AC is one of the widely used approaches in treating breast cancer, demonstrating comparable survival rates to radical mastectomy [14]. However, recent findings suggest that the likelihood of experiencing a local recurrence in breast cancer differs depending on the breast cancer subtype, as characterized by estrogen receptor (ER), progesterone receptor (PR), and HER2/neu status. Patients with TNBC exhibit higher recurrence rates when compared to those with non-triple-negative subtypes [15]. The objective of this study was to characterize a group of Jordanian triple-negative breast cancer (TNBC) patients who underwent either wide local excision with axillary clearance (WLE&AC) or modified radical mastectomy (MRM) along with adjuvant chemotherapy. Additionally, we sought to explore the clinicopathological distinctions between these two treatment modalities.

In this retrospective study, 110 women with TNBC were recruited with a mean age of 52. The prevalence of the triple-negative subtype in another Jordanian study was 11.7%, in which 42 out of 752 breast cancer cases had receptor-negative status and were less than 40 years of age [16]. These findings are also in line with international cohorts suggesting that TNBC prevalence falls between 10 and 24% of breast cancer cases [17]. Furthermore, invasive ductal carcinoma accounted for the most common histology (85%) of TNBC patients, followed by invasive metaplastic carcinoma in 14% of TNBC patients, while only one patient had invasive lobular carcinoma. Emerging findings regarding the clinical consequences of breast cancers with invasive ductal carcinoma (IDC) histology displaying the triple-negative phenotype suggest a highly aggressive disease trajectory and poorer clinical outcomes [18,19]. In a study by Liu et al. of TNBC patients with IDC histology vs. ductal carcinoma in situ showed that TNBC-IDC patients had a significantly worse disease-free survival (DFS) compared to TNBC-DCIS [20].

In our cohort, 25% of patients underwent neoadjuvant chemotherapy prior to radical mastectomy. Although there is biological diversity observed, the standard approach for neoadjuvant therapy typically involves traditional chemotherapy. The utilization of innovative targeted treatment approaches, like immune checkpoint inhibitors and poly-adenosine diphosphate ribose polymerase (PARP) inhibitors, is frequently restricted to cases of metastatic TNBC and is contingent on coverage provided by health insurance providers [21,22]. A systematic review and meta-analysis by Poggio et al. showed that in patients with TNBC, the use of platinum-based neoadjuvant chemotherapy is linked to an enhancement in pathologic complete response (pCR) rates, but at the expense of increased hematological toxicities [23]. Furthermore, the consideration of platinum-based neoadjuvant chemotherapy is a viable option for TNBC patients [23]. Furthermore, a comparative study by Xie et al. showed that the combination of MRM with neoadjuvant chemotherapy can yield favorable outcomes for breast cancer patients in various aspects, including reduced operation time, shorter hospitalization periods, decreased intraoperative bleeding, minimized postoperative complications, and improved quality of life [24]. It has been shown that patients with TNBC typically exhibit more favorable responses to neoadjuvant treatments compared to those with non-TNBC, and patients who attain a pCR following neoadjuvant therapy experience improved survival prospects [25,26].

Lymphovascular invasion was seen in 41% of TNBC patients in our study and was associated with a significantly higher lymph node positivity and LNR. LVI is well-established to be an important prognostic marker for breast cancer, and in a prior study by Schoppmann et al., it was documented that lymph-vascular invasion (LVI) stands as an autonomous prognostic indicator for both DFS and overall survival (OS) in individuals diagnosed with breast cancer [27,28]. In addition, a study by Ahn et al. on non-metastatic TNBC patients who underwent surgical resection showed that LVI was significantly associated with tumor relapses with 58.3% 3-year disease-free survival [29]. Consequently, there is a pressing need for the development of more efficacious treatment approaches for TNBC patients presenting with LVI [29].

Our study provides several strong points. Our study addresses an important clinical issue by investigating the characteristics and treatment outcomes of TNBC in a population with limited existing research, specifically in Jordan and the Middle East. This can provide valuable insights for healthcare professionals in the region. However, our findings should be interpreted with caution due to several limitations. First, we had a limited sample size, which might limit the generalizability of the findings. In addition, our study was retrospective in nature, which may not allow us to investigate disease progression over time. Future longitudinal studies are warranted to track TNBC patients over time, allowing for a better understanding of the disease's progression and treatment outcomes.

## Conclusions

In conclusion, our retrospective analysis sheds light on the clinical characteristics and management of triple-negative breast cancer (TNBC) in the Jordanian population. The study aimed to fill a crucial gap in the existing literature regarding TNBC in Jordan and the Middle East. Our findings reveal that TNBC patients in Jordan, who underwent breast-conserving therapy or mastectomy along with adjuvant chemotherapy, exhibited clinicopathological variations. Notably, we observed a higher prevalence of invasive ductal carcinoma, emphasizing the aggressive nature of TNBC. The utilization of neoadjuvant chemotherapy, particularly platinum-based regimens, and the association of lymphovascular invasion with higher lymph node positivity underscore the complexity of TNBC management. Despite the study's strengths in addressing a significant clinical concern and providing insights for healthcare professionals in the region, its limitations, including modest sample size and retrospective design, should be acknowledged. Future longitudinal studies are warranted to delve deeper into TNBC's progression and treatment outcomes in the Jordanian context. These endeavors will contribute to a more comprehensive understanding of TNBC dynamics and aid in refining therapeutic strategies for better patient outcomes.
